# Adaptable data management for systems biology investigations

**DOI:** 10.1186/1471-2105-10-79

**Published:** 2009-03-06

**Authors:** John Boyle, Hector Rovira, Chris Cavnor, David Burdick, Sarah Killcoyne, Ilya Shmulevich

**Affiliations:** 1 Institute for Systems Biology, 1441 N 34th Street, Seattle, WA 98103, USA

## Abstract

**Background:**

Within research each experiment is different, the focus changes and the data is generated from a continually evolving barrage of technologies. There is a continual introduction of new techniques whose usage ranges from in-house protocols through to high-throughput instrumentation. To support these requirements data management systems are needed that can be rapidly built and readily adapted for new usage.

**Results:**

The adaptable data management system discussed is designed to support the seamless mining and analysis of biological experiment data that is commonly used in systems biology (e.g. ChIP-chip, gene expression, proteomics, imaging, flow cytometry). We use different content graphs to represent different views upon the data. These views are designed for different roles: equipment specific views are used to gather instrumentation information; data processing oriented views are provided to enable the rapid development of analysis applications; and research project specific views are used to organize information for individual research experiments. This management system allows for both the rapid introduction of new types of information and the evolution of the knowledge it represents.

**Conclusion:**

Data management is an important aspect of any research enterprise. It is the foundation on which most applications are built, and must be easily extended to serve new functionality for new scientific areas. We have found that adopting a three-tier architecture for data management, built around distributed standardized content repositories, allows us to rapidly develop new applications to support a diverse user community.

## Background

To enable the adaptive behaviour that is required when developing software for research an "informal" data management strategy is often needed. By informal we mean there is a need to rapidly develop and adapt software infrastructures to unforeseen and (typically) unspecified requirements. We have found that the use of a distributed data management system (consisting of remote interlinked content repositories) gives us the required flexibility, while still allowing for the development of the level of formalization that is required for robust software development.

Advances in computer science have pushed what can be achieved with data management systems, and conversely these advancements have driven the increase in demands for richer functionality. The computer science research advancements have involved both hardware and software, with faster processor speeds enabling other innovations to become feasible. The way in which data management systems are built, and extended, has also changed. These changes in software engineering and design include: the *methodology *through which software is constructed (e.g. components leading to frameworks, and frameworks leading to aspects [1]); the *technology *used to allow for distributed computing (e.g. object brokers evolving pass-by-value mechanisms, and these being replaced by stateless Web Services); and the *ideology *that is used to define the process through which software is built (e.g. the "rational" processes being replaced by agile programming). These advances are continuing to occur, and will have an effect on the next generation of data management and distribution tools (e.g. cloud computing becoming mainstream through the use of Google App Engine or similar).

**Figure 1:**
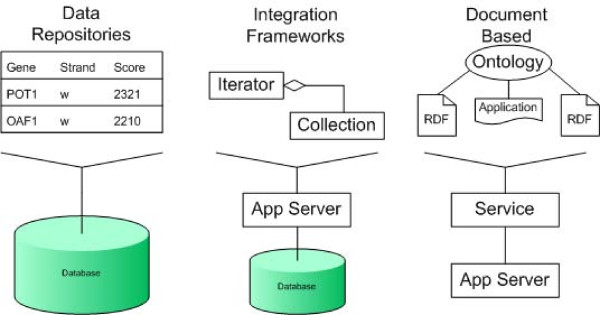
**Evolution of enterprise architectures has occurred within the life sciences**. Limitations in the flexibility of data repositories based solutions helped shape the development of integration frameworks. Integration frameworks suffered from complexity and interoperability problems, and so document based solutions are now becoming the norm.

A number of companies, and academic institutions, have marketed integration and data management solutions for the life sciences. These enterprise data integration (and distributed process) management systems have evolved over the last 10 years. This evolution has been from single database based solutions to open, distributed, interoperable data management solutions (see Figure 1). This change has been driven by demands for rapid development, high levels of interoperability and increases in data volume and complexity. There has been a natural progression with these integration systems, as they generally follow the traditional approaches to software designs and technologies that are prevalent at the time. There are numerous examples of the application of technical innovations being the focus of a specific integration product, for example: in *1996 *SRS [2] (from Lion Bioscience) advocated external indexing to link between numerous gene and protein data sources; in *1997 *the Discovery Center (from Netgenics) used CORBA [3] based distributed components to provide bespoke integration products; in *1998 *the Alliance framework (from Synomics) promoted an n-tier application server distributed system, which used linked domain specific modules; in *1999 *the MetaLayer (from Tripos) utilized XML message passing; in *2000 *DiscoveryLink [4] (from IBM) provided a federated database solution which linked across different databases and flat files; in *2001 *the Genomics Knowledge Platform (from Incyte) marketed an object integration solution based solely on EJBs; in *2002 *the I3C (a consortium led by Sun and Oracle) specified the use of an identity driven approach to integration; in *2003 *the LSP (from Oracle) advocated the use of Web Services; in *2004 *IPA (from Ingenuity) and MetaCore (from GeneGO) used a knowledge base to provide a solution for the mining of networks of integrated data; in *2005 *caBIG [5] (from NCI) adopted a MDA (model driven architecture) approach, built using a J2EE and Web Service based solution, to standardize their community integration efforts; in *2006 *CancerGRID (from MRC) delivered a resource framework based Web Service system to bridge between diverse data sources; and in *2007 *caGRID [6] (from NCI) provided a stateful Web Service and registry system for loosely coupled data and analysis services.

One common characteristic of these "technology first" efforts is that they developed solutions that were designed to work with static "finished" data, not research information. This style of system works well within publishing scenarios, where information is to be made available throughout an enterprise as a static resource. When actually working within a research institution (or life science company), where new technologies and ideas are continually being developed, such static publishing approaches are not appropriate. Instead, a flexible analysis and access system is required that allows for the rapid introduction and integration of many types of data. With the advent of systems biology, the recognized need for integration solutions has reached a high level of urgency.

This article is principally focused on the design of software to support systems biology investigations, rather than a discussion of a specific application. The software that has been developed, and made available to the community, is discussed to illustrate the advantages of the advocated designs. The importance of design can be over looked in scientific computing [7], due to the complexities with the development of software for research which arise due to the constantly changing requirements and individual project-centric approaches. In this article we discuss how we designed a flexible and maintainable data management solution which can be readily adapted to meet the requirements of a complex research environment. The reasoning behind this non-traditional design is that a research software infrastructure must be highly adaptable to change. The required change could arise from new technologies being introduced, a change of research focus, or a change in resourcing (project funding). How we integrate the data management system with other tools and data sources has been discussed previously [8].

## Results

There are two major requirements on software design within a rapidly changing research environment: the ability to develop and integrate software rapidly, and the assurance that the resulting systems are adaptable to new and unpredictable needs. The methodologies and technologies used within research environments must be able to satisfy these requirements. Methodologies that depend on formalized specifications for processes and data structures are not appropriate for research software infrastructure. An understanding of current informatics technologies, and their suitability, is essential to ensure that research driven software projects can be delivered on schedule. Over the last few years there have been many advances in enterprise computing which means that data management technologies can now effectively be used in rapidly evolving research environments. These advances mean that, through an appropriate choice of technologies, it is now possible to deliver, within a minimal timeframe, a distributed system which is robust, standardized, loosely coupled and interoperable. Enterprise components can now be rapidly configured to deliver a range of rich functionality (e.g. content management, messaging, state based processing, dynamic discovery, high level orchestration). However, inappropriate technology choices can result in an architecture which is not adaptable and difficult to maintain.

Technologies that require a static structure (e.g. schema definitions) are constrained in their usage as they require "top-down" design. This means that the usage of traditional application server based technologies may not be appropriate for many aspects of research, as they are largely designed for tasks where: there exists reasonably stable information which can be structured in a DBMS or similar; business logic is required to operate closely on the data; or integration logic or data transformation is required. Alternatively, less structured data management, such as content repositories, can be suitable within research environments. Content repositories serve a different purpose to application servers, but can be used to serve out similar types of information using the same protocols. Content repository technologies can be more appropriate (than an application server) as they provide a high level of flexibility when dealing with unstructured content (e.g. experiment information).

We are not suggesting that more formalized representations of data do not have their place in research environments. Many data sources can be represented as static snapshots, and when it comes to presenting the research results, the use of more standard software development practices and tools is essential. These static approaches become unsuitable in situations where software is being developed to directly support on-going research projects, with unpredictable life cycles and unforeseen requirements.

### Layered content management

**Figure 2:**
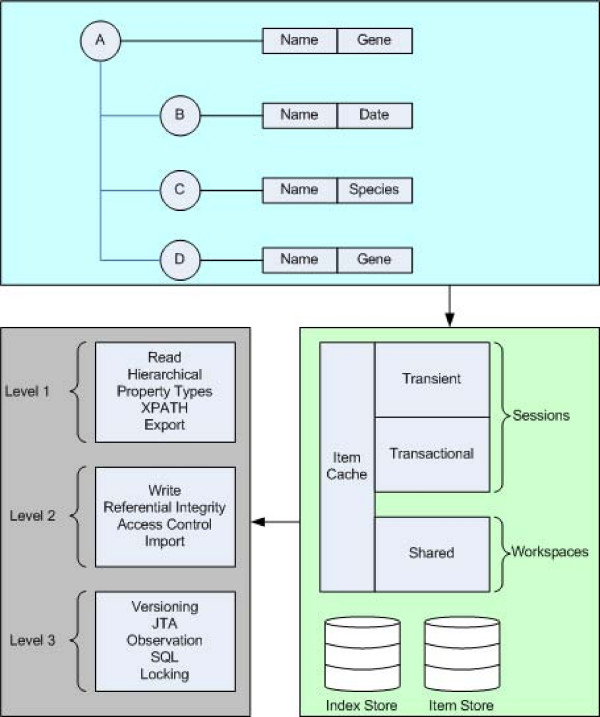
**Content management systems are designed to handle generic data items**. These items are placed within a hierarchical structure. Each session with a content repository is managed, so that changes can be made in a transactional manner. A separate indexing service can be used to enable searching through annotations attached to items. Generally three levels of functionality are supplied with content managed systems: level 1 refers to "read" based operations; level two is for "write" based operations, such as maintaining referential integrity and providing access control; and level three is additional operations such as versioning, observation/notification and concurrency.

To manage data arising from ongoing research experiments we adopted an approach using distributed content repositories. Content repositories allow for the development of a formalized structure that can be associated directly with resources (see Figure 2).

The content can be organized into a (typically) hierarchical structure. The nodes in the hierarchy can have an arbitrary complexity (depending upon the requirements) and also have extensible property sets associated with them. The content system also provides for the usual array of horizontal services (e.g. data querying, browsing, management, transactions, concurrency control, security).

We extended the Java Content Repository (JCR) standard (using the Apache Jackrabbit implementation [9]) to provide a distributed data management system that consists of a series of interlinked content management systems (CMS). Such a federated content repository solution offers three major advantages over a standard database/application server solution:

• ***Easy to adapt***. Within a research environment it is generally difficult to hammer down requirements. The requirements change over time, and new functionality is often required at short notice. Content repository systems have a high degree of flexibility, as the content graph can be extended to meet new requirements, and the annotations can be dynamically updated. This means that when new requirements become apparent, the structure can be changed or modified easily, without having to rewrite a schema or change an object layer implementation. It is interesting to note that other disciplines, in particular the design community, have found that such flexibility is invaluable in enabling creativity [10].

• ***Easy to understand***. Any data management solution will involve a high level of complexity, especially in a distributed research environment. This complexity, however, does not mean that the principles of how the system operates cannot be easily understood. Allowing the end-users to build up a good mental model of how the system works removes many obstacles with adoption. By portraying the content management as "an intelligent file system" positive transfer of knowledge can occur, so that the system is natural and intuitive to use.

• **Easy to access**. Within a research environment there is little time to be spared for learning (largely transient) informatics systems. This fact coupled with the low level of formal software training of most researchers (whether computationally inclined or not), means that convenient access to the data is of paramount importance. Content management systems in general, and the system discussed in this paper in particular have little complexity in terms of object models and data access protocols, as the storage structure is simply a hierarchy and a large number of access protocols can be supported (e.g. direct file I/O, RMI, WEBDAV, REST).

Unfortunately, a single content repository system does not offer the level of flexibility that is required. Research groups interact with different facets of research information in diverse ways and with a plethora of goals. This diversity means that the data must be organized in different ways, for example: when capturing the data it may be appropriate to organize information by group and date; when processing the information it can be organized by runs and tool information; and when exploring the information it can be organized by biological significance (e.g. by gene, by strain, by condition).

**Figure 3:**
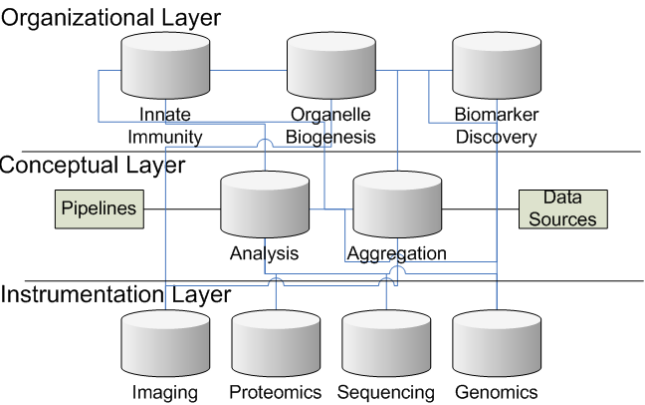
**Schematic diagram showing how loose coupling between content repositories has been used to provide a distributed content management system**. The data management system allows for the ad-hoc linking of items between repositories, using URIs. This allows for the simple linkage of items between any of the repositories. Generally laboratory scientists generate data and organize it directly within the "instrumentation layer". Once the data is available pipelines are built which query and retrieve experimental data from the instrumentation layer, the subsequent analysis results are stored in the analysis layer (including any logging information and intermediate results). The aggregation system allows for indexing of large data files so that subsets of information can be retrieved. The organizational layer allows for the results of analysis (or sub sets available through the aggregation system) and raw experiment results to be combined with additional research information.

To allow for this diversity (and adaptability) we deployed a series of loosely coupled distributed content repositories. The resulting distributed content management system (Figure 3) allows for different people to interact with the systems directly. To identify resources we have previously [8] used URNs (encoded as LSIDs). We have since migrated to using HTTP URIs, as we feel that HTTP URIs have advantages over URNs for linking between data sources: URIs (like URNs) can support human readable hierarchical naming schemes; Representational State Transfer (REST) operations can be easily appended to URIs; they do not require additional overhead for resolution; and URIs are better aligned with current community efforts.

The main aim of the distributed content repository system is to enable the researchers, and associated research tools, to easily be able to store, manipulate and retrieve the information they want in the form they desire. One repository can span multiple installations, and multiple repositories can reside within one instance. The system we use is designed around a three-layer architecture:

• Instrumentation Layer. This layer is used to capture experimental information. The layer models information in a way that makes storage of the information simpler, so that laboratory scientists can easily add and annotate information that is captured from a variety of instruments. Typically each type of experiment has a distinct (and differently structured) repository.

• Conceptual Layer. This layer is designed to provide a means to generically interact with the information through the use of high level abstract operations. These operations include the aggregation and retrieval of information, and do not necessitate an understanding of the actual information content. Information which is required for manipulating the data is provided as metadata through properties attached to the data files.

• Organizational Layer. This layer provides a project (or researcher) based view on the information, and therefore is designed to have a "biological focus". Typically the content is organized by factors such as disease, organism or molecule. Each different research, or research group, can individually organize and annotate the data to suit their individual requirements.

**Figure 4:**
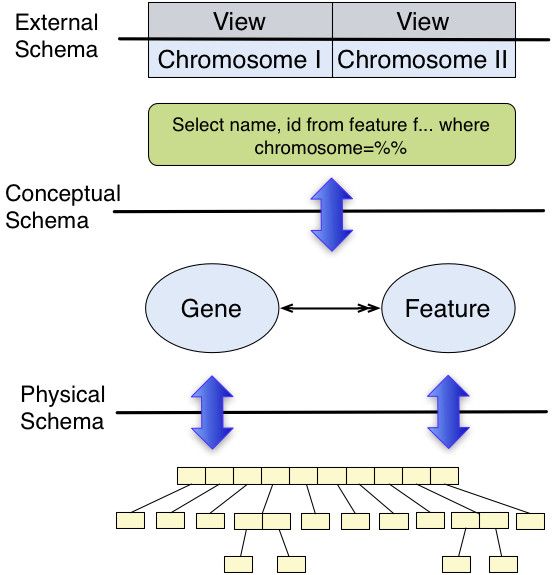
**The ANSI-SPARC three layer database architecture was proposed in 1975, and is still used in modern RDBMS**. The three proposed layers were a physical schema which defined how the data is actually stored (inode information), a conceptual schema which represented how information was related and indexed, and an external schema which represented how information was presented. The architecture was designed to provide immunity to change: the physical schema defined how the actual information was stored, and could be changed without effecting how external applications interacted with the data; and the external schema could be changed to define richer APIs, without having to change the underlying storage mechanism.

**Figure 5:**
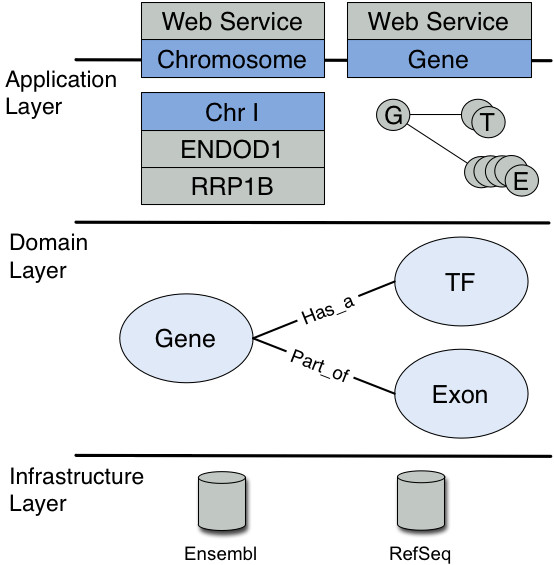
**Design of a system within an application server typically involves three layers: an infrastructure layer which stores the data; a domain layer which is populated with vertical object models; and an application layer which models how an application can interact with the domain objects**. The application server provides both resource management and service based functionality for each of these layers. In EJB terms there is: the infrastructure layer corresponding to a database, typically with O-R mappings through hibernate; the domain layer corresponding to entity beans, which basically function as an object cache; and the application layer corresponding to sessions beans or Web Services (with stateless session bean mappings).

It is interesting to note that "three layer" architectures commonly occur in many software architectures [11]. This pattern consistently has a lower level that directly reflects the underlying data storage, a middle layer which provides an abstract conceptualization of the data, and an upper level which presents the data in a convenient manner to external applications (see Figures 4 and 5).

### Instrumentation layer

The instrumentation layer is designed to be the interface point with high throughput equipment and laboratory instrumentation. Data is captured by laboratory scientists via equipment and is stored in a repository that is structured for their convenience. In this way the repository is closely aligned to the experiment and experimenter.

The repositories are mainly used to automatically capture information from high throughput experiments. We are currently using instrument repositories to capture information for high throughput imaging, proteomic and genomic experiments. The instrumentation repositories generally have relatively simple structures as their purpose is to facilitate data collection. For example, when capturing information for genomics, the data is organized in the repository in a fairly flat hierarchy containing a simple name and time stamp mechanism.

We specify a limited set of properties (based upon an ontology with an associated controlled vocabulary) which are used to describe the metadata associated with the experiment. This controlled metadata is used in conjunction with any ad-hoc or free text annotations which may be added at a later time by the experimenter. Where possible the metadata is gathered directly from the instrumentation (e.g. by parsing data files generated by microscopy control equipment such as IPLAB) otherwise, we interface with bespoke sample tracking systems (e.g. for genomics we bridge with the sample tracking SLIMArray [12] software). The ontologies used are based upon community standards (e.g. OME [13], MAGE [14]).

Once the data is collected within a repository the content can be queried, browsed and retrieved using standardized mechanisms, such as: object protocols (e.g. RMI) to develop distributed systems; WebDAV to provide document-based browsing (and mappings to file system browsing tools); XPATH query for structured and unstructured searching/retrieval; and REST/JSON protocols for programmatic access from any language that supports HTTP connectivity (e.g. Java, Matlab, R, JavaScript, Flash). To meet our needs we have also provided two additional retrieval mechanisms:

• URI based retrieval – all referenceable items within the repository can be retrieved using a HTTP URI. This interface was built both to provide convenient access to the data and also to allow for the construction of semantic web based applications on top of a JCR instance.

• Standardized access components for retrieving, searching and publishing information to a JCR instance. We have used these components in a variety of applications, including: within a servlet based web application; within a standalone application(s); and as part of a GenePattern [15] tool set.

### Conceptual layer

The conceptual layer is designed to aid in tasks that are fundamental in many different research investigations. The layer provides a high level of abstraction for tasks associated with data processing (the "analysis system") and data manipulation (the "aggregation system"). These systems provide common (horizontal) functionality, and are designed to be used by analysis and data processing subsystems.

#### Analysis Subsystem

**Figure 6:**
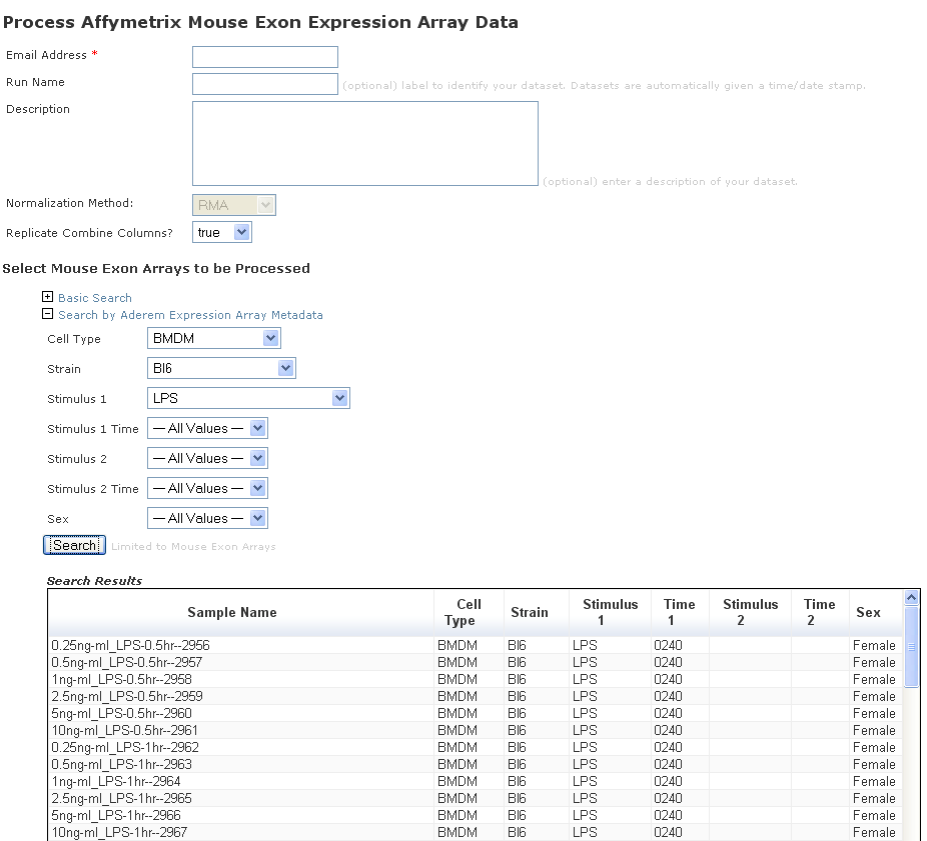
**Example view on a gene expression array normalization and analysis pipeline web application**. This web application is used to normalize sets of gene expression experiments together so that they can be compared. The researcher can use a variety of search tools to select the arrays they wish to use: basic search performs a string match against any annotations in the content repository and the search by meta data is used to perform exact matching of names against node properties. Data is retrieved from the instrumentation layer and a new node is created in an analysis repository which represents the specific request. An analysis pipeline is then started using information stored in the analysis node. In this case the analysis pipeline is built within Gene Pattern. As the run progresses all status information is passed to the analysis node, so that the run can be monitored by an administration application. When the run is complete, the final results are also stored in the repository, and this triggers a notification event (typically an email) to be sent.

**Figure 7:**
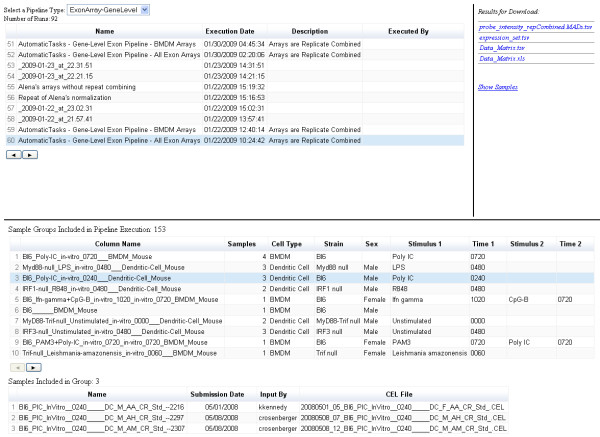
**Administration and inspection tools can be built against the analysis repository, which show information about specific analysis that was undertaken**. These tools simply display the information about completed analysis runs. They can be searched, and the results fro each analysis can be retrieved. The above tool shows a view on the analysis repository that is used for all gene expression analysis runs, the type of chip is chosen using the top drop down menu, and then information about each analysis (e.g. descriptions of the chips that were analyzed) is shown. The federated repository allows for loose coupling between the web applications, administration tools and the analysis pipeline framework.

The analysis system is used to manage, audit and store the results of data analysis/processing pipelines. Each pipeline run is associated with a node in the analysis content repository. Each node defines a structure that accommodates the required inputs, the outputs and logs produced by the process, and the current status (and history) of the execution. In this way, each processing pipeline is loosely coupled with the analysis system, which provides a searchable storage area for input/output files and a logging service. Other software (e.g. administration/monitoring applications) can query the repository to find the status of any distributed processes that has been registered. A process that uses the analysis system typically has the same life cycle: when a processing pipeline starts it queries the content repository to find the required input node (the querying is based upon the properties that have been set on the node); status information is sent to the status node (e.g. state transitions, failures, logging); and output data is stored in the output nodes. Due to the loose coupling and simplicity we have used the analysis system to support numerous data processing pipelines, in particular in processing genomics data (see Figures 6 and 7).

The genomics normalization pipeline (Figure 6) uses the analysis system directly. As the analysis system uses a standard pattern of nodes (input, output and status hierarchies), any analysis pipeline can make use of it. The genomics normalization pipeline simply retrieves the required input parameters, executes the job (and updates the status), and then writes the results. By using a pattern of nodes, applications can be loosely coupled so that they can be developed independently of each other with few dependencies e.g. a job submission application can write to the input nodes (Figure 6), and a separate processing application can receive notifications that are triggered through changes to the status nodes (Figure 7). The loose coupling provides a high level of reliability and allows for rapid application development. The content system also provides for a means to ensure that we are able to achieve reliable auditing of each analysis run. The basic functionality for reading/writing information is provided through standardized modules.

#### Aggregation Subsystem

One of the aims of the computing advances over the last few years has involved the concept of *run time aggregation*. This approach is epitomized by the semantic web, where people can mash information from a variety of data sources into a single graph. We provide a run time aggregation system specifically for the aggregation of data from different analyses.

**Figure 8:**
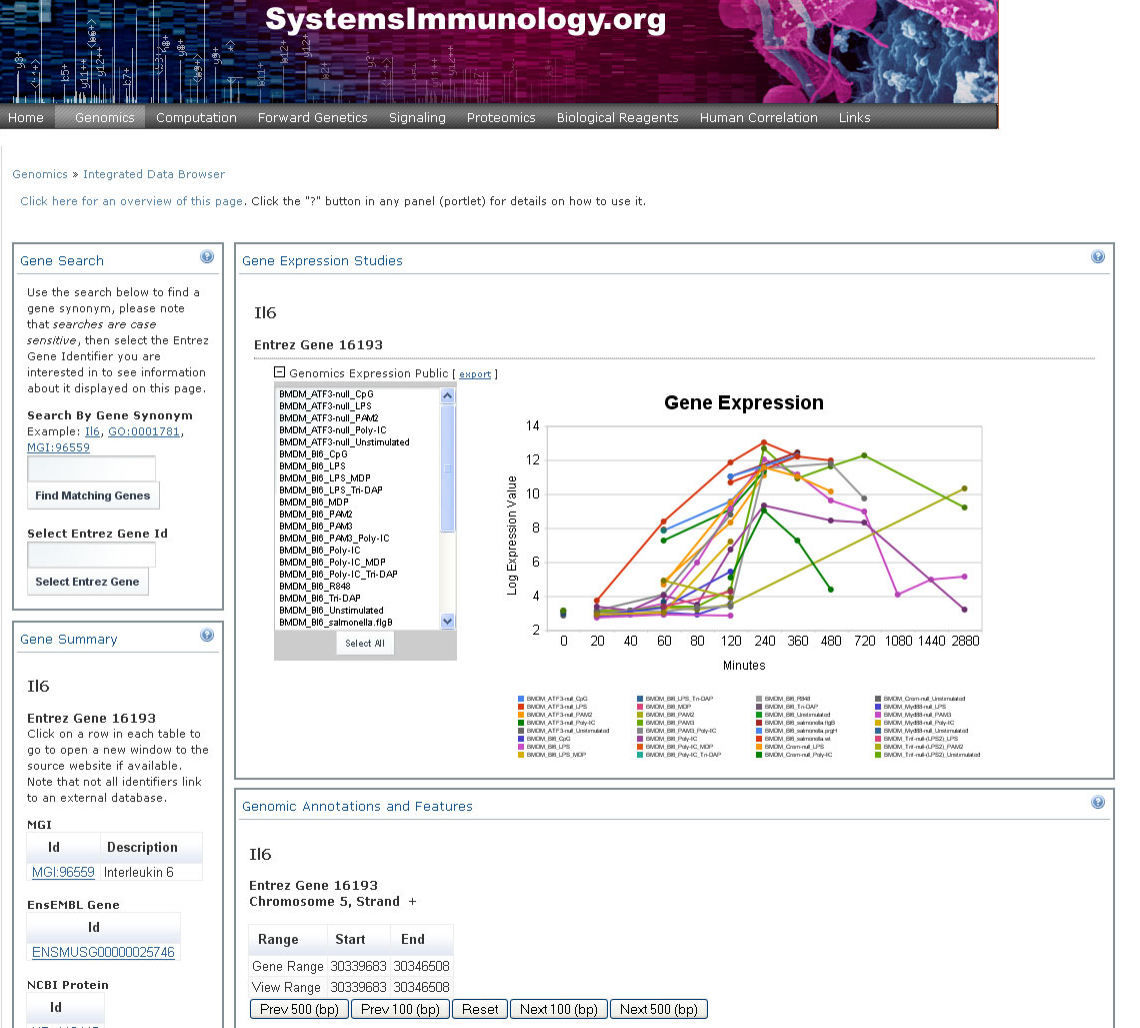
**The above example Web Application uses a portlet engine to display information from a variety of repositories (and other data sources)**. The aggregation system can be used to pull together information from different sources. It can be used directly to index and then extract subsets of information from larger data files. The above example web application is a prototype application which uses our adaptive data management system to store information about gene annotations, ChIP-Chip experiments, transcription factor binding site predications and gene expression studies. The Gene Expression Portlet transparently accesses and extracts subsets of information from data files by simply querying the aggregation system by gene name.

When an analysis run has finished, the observing aggregation system can trigger an indexing operation on the results. The indexing is controlled using the properties that are associated with the output of the analysis run (typically the properties related to the rows or columns that are to be indexed). As all data within any of the content systems is indexed it is possible to retrieve data items using free text or direct querying. If, for example, an application (or user) wished to aggregate all data pertaining to a specific gene then it can query the aggregation subsystem (and other content repositories) so that the aggregated information retrieved would consist of both nodes (representing both specific data and any associated metadata) and specific rows (or columns) from data files. In this way, it is possible to extract relevant information from a variety of data sources upon demand. For example, the end results of the normalization pipeline shown in Figure 6 is a large replicate combined data matrix of how mouse gene expression levels change over a myriad of conditions; the aggregation system can be used to automatically extract subsets of this information directly so that they can be presented in a uniform manner (see Figure 8).

The Web Portal (Figure 8) uses the standardized service to retrieve information from a number of repositories. The service can query multiple repositories, so that the portal makes a request to retrieve URIs for specific types of information that match a specific gene id. This information is then displayed in each of the portlets. The aggregation system is used to allow for each part of data files (e.g. a specific row in a matrix that corresponds to a particular gene/probe) to be uniquely identified by URIs.

### Organization layer

**Figure 9:**
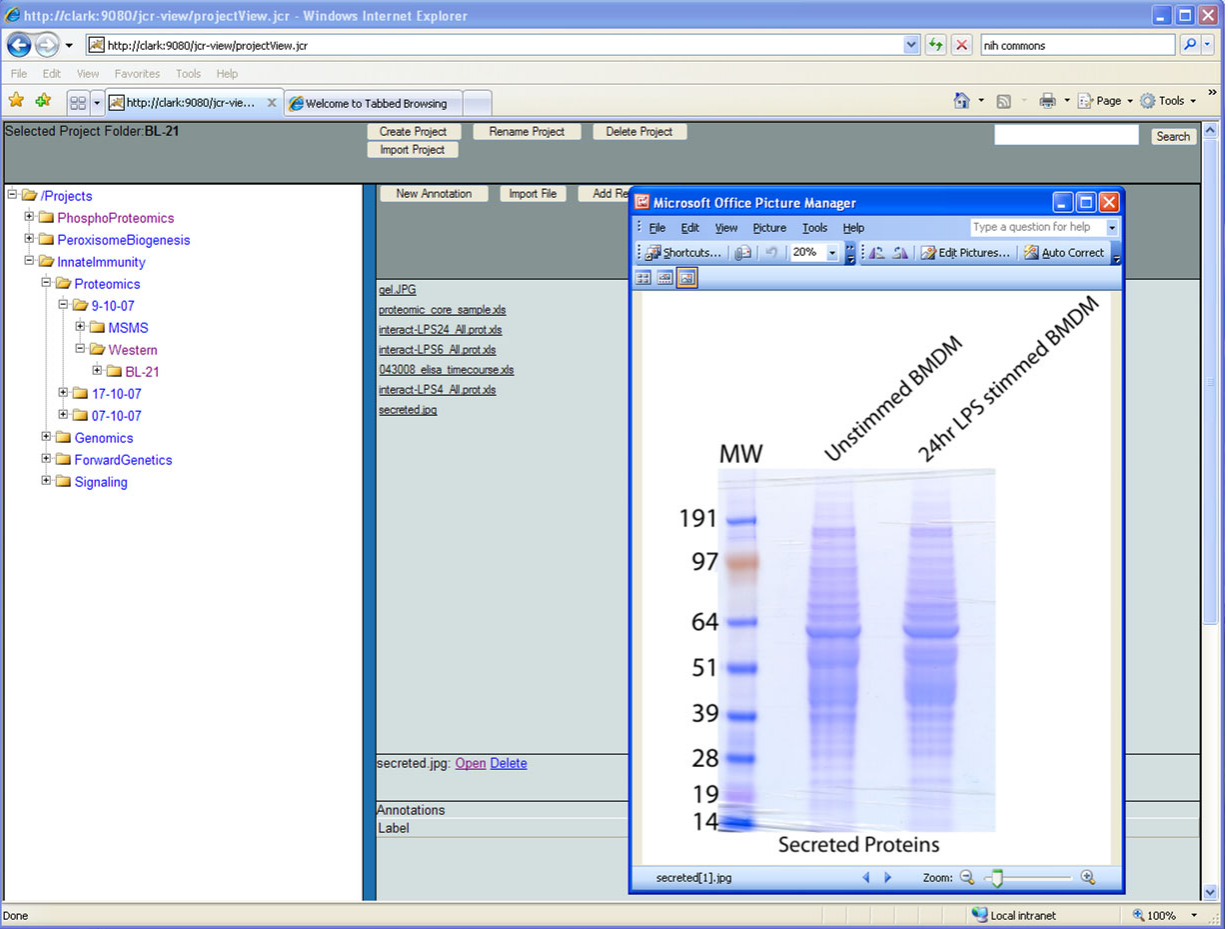
**Purpose built Web Application for browsing distributed repositories**. Although there are a variety of tools available for browsing single site (and single workspace) repositories, the above application allows for the transparent linking and browsing of distributed repositories. In this example, a repository has been overlaid on the other repositories which provide a project structure on the information.

The organization layer allows for the construction of a scientific oriented façade on top of the other repositories. This façade provides a scientific project based structure, which allows for the custom organization of data and for project specific annotations to be added. This layer provides more than a materialized view of the other layers, as extra information and data relevant to the specific scientific project can be added, searched and retrieved. As all "referenceable" items within any of the repositories are exposed as HTTP URIs, these can be used to provide links between distributed repositories. As all items are uniquely addressable, a generic browsing application can be built (see Figure 9) which provides a view on the interlinked repositories. The browsing application can be used to annotate items so that they can be searched, to organize distributed items, and to link items from different repositories.

**Figure 10:**
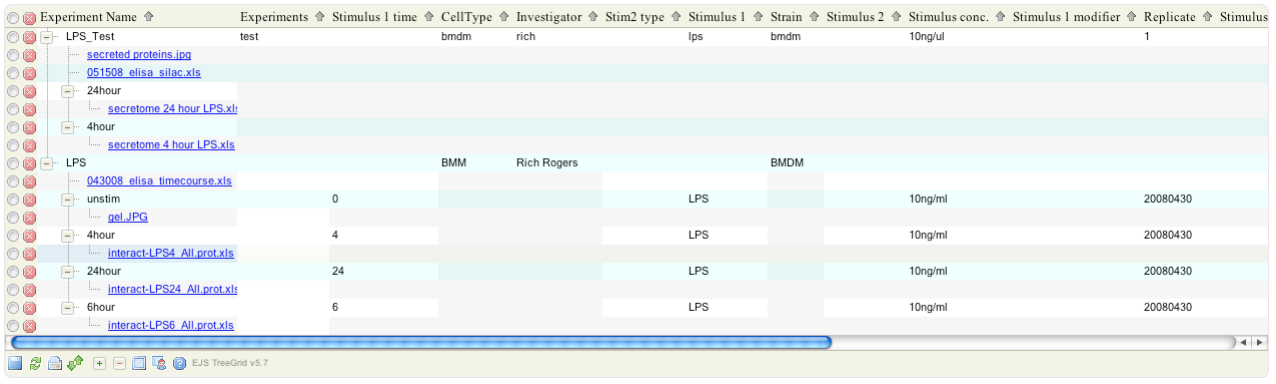
**Proteomics data browsing web application**. This is an example of a web application that has been constructed on top of the organizational layer, the application allows for browsing/sorting by annotations and linking of experiments relating to a specific tandem MS experiment (e.g. western, ELISA, etc). As rapid application development and easier maintenance are essential in fast moving research environments, the underlying repository is structured to direct reflect how the proteomics experimenters wish to interact with their data. This structure is effectively mirrored in the web application, enabling rapid development and reducing transformation complexities.

The endpoint for URI addressed items is a REST Web Service implemented as a series of servlets. These Web Services are used to provide interoperable operations on both individual repositories (see Table 1) and items within a repository (see Table 2). Any domain model representations (in Request and Response) are formatted using JavaScript Object Notation (JSON). The JSON syntax is fast becoming a standard for communications over REST APIs as: it is lightweight; easily extensible and human readable; is easy to interpret from many programming languages; and can conveniently be transformed to other formats such as XML. We use this API to rapidly develop applications for specific users or research groups on top of repositories that (generally) directly reflect the "mental model" that the users have about the organizational structure of their information. An example of how we have used this layer is an application that is used to organize the results of proteomics experiments (see Figure 10). This application simply mirrors how a research group used to organize their data locally (e.g. the directory hierarchy they used to store gels, proteomics results, ELISAs). The application has richer functionality as the items can now be annotated, is accessible (and versioned) and the relationships between items can be browsed (including access to the raw data files).

**Table 1:** Repository level operations that are provided through http encoding.

**Operation**	**Base ** **Syntax**	**Description**
Browse	/<XPATH>	Providing a repository PATH parameter (e.g, /analysis/runs) returns a generic representation of the underlying resources.

Create	/new	Create new resources by appending '/new' and providing parameters for a repository PATH and a resource NAME. Files can be uploaded as part of a multi-part HttpRequest.

Search	/search	Search by query terms by appending '/search' and providing parameters for a repository PATH. Query terms are matched against underlying resource properties (e.g. Organism = Mouse).

**Table 2:** HTTP URI based operations are provided through a REST API for operating on individual objects.

**Operation**	**Base Syntax**	**Method**	**Description**
Retrieve	/<XPATH>/meta	GET	Retrieve JSON representation of a resource, and an array of its child resources

Retrieve	/<XPATH>/dir	GET	Retrieve a file system representation of the resource with directories and files.

Retrieve	/<XPATH>/structured	GET	Retrieve the domain model representation of the resource.

Search	/<XPATH>/search	GET	Retrieve underlying resources matching provided query parameters.

Modify	/<XPATH>	POST	Updates domain model representations, properties, files or contents of a file.

Delete	/<XPATH>	DELETE	Permanently removes the resource, its model, properties and files

**Figure 11:**
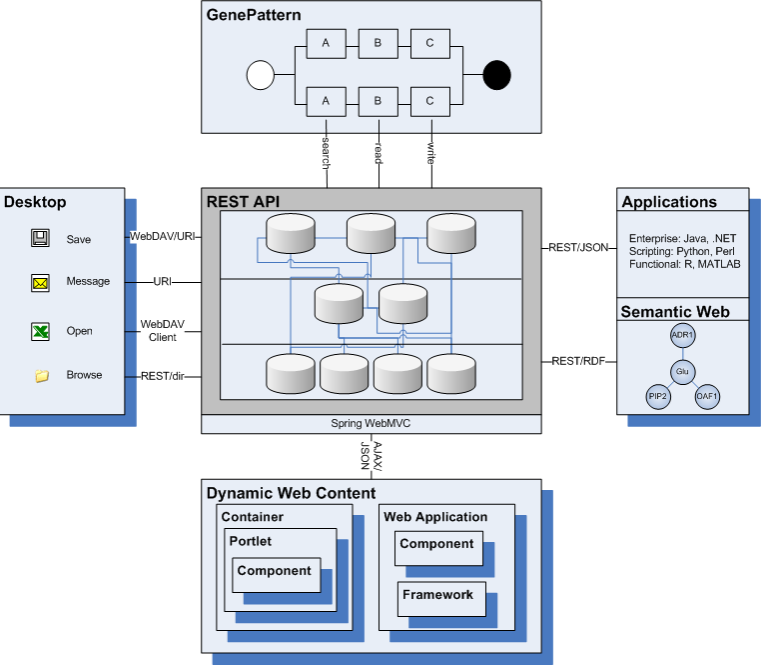
**The use of communication standards allows for a high degree of flexibility in delivery of information**. Data stored in files can be either imported or linked into the content repositories, and then annotated. RMI interfaces expose the basic read/write functionality. This information can be searched using structured querying (sql or xpath based), as well as using free text (inverse index based) searching. To ensure accessibility to the data and flexibility of use we provide a REST API which provides additional integration points. For the data analysis pipelines (or toolsets in GenePattern) standardized modules are provided which use the REST API directly to search for files, as well as read/write information into the content repositories. Application programming language integration is provided through the REST API returning JSON objects, so that while the integration is stateless it supports any language that can access URIs and parse JSON (e.g. Matlab, R, Java, C#, Perl). The system can also be used to integrate with the semantic web, as RDF graphs which directly represent information within the repository can also be returned. Dynamic Web Content is generated and managed using Spring, with controllers being used to service AJAX requests. We use AJAX/JSON calls to populate pages and visualizations in a variety of web frameworks (including portlets and JSF). Desktop integration is essential to the usage of the repositories, and the REST access layer can be used to return information in a number of protocols: URI access can be used to allow direct access; WebDAV can be used to integrate with common desktop tools and allow for browsing; and the REST API can be used to return directory (tree) based structures.

The organizational repositories represent a domain-oriented view on the information. The information can be securely retrieved using a variety of protocols (see Figure 11). The flexible access to the data means that: scientists can browse, arrange and annotate the information using a hierarchy that best suits their needs; and domain-centric applications can be rapidly developed using these structures as interfaces to the data.

## Discussion and conclusion

The adaptive data management system we have discussed has been designed to meet the competing requirements for data management that arise within a research organization. As with many aspects of research informatics, flexibility and rapid development are key issues as requirements change unexpectedly and frequently. We advocate the view that, to be of lasting use to research, any data management system must be able to:

### Support the evolution of ideas

The development of integration strategies for systems biology is problematic due to both the nature of science and the organization of scientists. It is typical that the means to which a specific experimental technology will be used, and the methods used to analyze the resulting data, is unknown at software design time. This is because scientific understanding continually evolves. Any scientific data management system has to support such evolution, meaning that traditional approaches to development and design are frequently inappropriate. As neither the data usage nor analysis is known a priori an easily adaptable solution is needed.

### Allows for flexible working

There is a fundamental requirement for scientists to easily access, query and manipulate data to suit their needs. Science led investigations require an infrastructure to support ongoing data driven discovery processes. This means that: the data should be accessible through a variety of mechanisms, including multiple computer languages and applications; the data should be detached from the system, so that scientists are not "tied down" to any specific object model or way of working; and flexible navigation through the data using a variety of approaches should be supported.

### Delivers the salient information

Many aspects of biomedical research can be thought of as an information centered science [16]. Information must be established in the context in which it has been requested, that is to say, the *view *on the information changes depending upon the question that was asked. Therefore, the structure of the data management system must change depending upon the requirements. In a research environment, the views on the data typically involve: high throughput instrumentation, where information must be pushed from machines quickly and reliably; computational informatics processing applications, which require auditing and annotations; and research projects, which require domain oriented and flexible views upon the data. This requirement for adaptable data management is encountered in many avenues of research across all of the biomedical sciences.

Rather than build a *de novo *system, we have extended standardized open source systems. By extending community standards we are able to deliver production systems quickly and are able to overcome boundaries due to resource limitations. We have found that the standardized systems we have adopted are generally not directly suitable for research informatics, and therefore had to be extended to allow for the required flexibility. These extensions are designed to: ensure that the system integrates well within a research enterprise; allow for customization of the system to ensure it has a richer semantics; and provide workflows to ensure the system can work robustly with high throughput instrumentation.

We have found that the three-tier distributed data management system provides the flexibility and adaptability that we required. As we adopted and extended standards, the system was put together with a minimal of FTE effort, while still providing a robust and scalable architecture. Most importantly, as science moves along at a rapid pace, and opinions are always multi-faceted and divided, this system allows us to evolve the structure of the data management to meet new requirements without having to continually rewrite or wrap out of date or inappropriate legacy code.

## Availability and requirements

We have made a number of tools available to the community. These tools are built upon the Web Services we have developed that allows for the interoperability and distribution of content across a number of JCR instances. For these tools to function one or more JCR compatible instances must first be installed, and then the REST services must be deployed within tomcat. Further instructions are given below, and more details (including the relevant template files) are given on the main download site.

## Project information

*Project home page*: ;

*Operating system(s)*: Platform independent;

*Programming language*: Java; Other requirements: Java 1.5 or higher, Tomcat 6.0 or higher, working JCR instance 1.4.0 or higher.

*Licence*: Apache;

*Any restrictions to use by non-academics*: no restrictions.

## Installation instructions

Detailed instructions on how to install the main data management system, which consists of the REST services and the project explorer web application are given with the downloads section of the project web site. To install the main software the following steps must be undertaken:

1) Ensure you have Java 1.5 installed and a working Tomcat (6.×) instance

2) Download the main services file (the addama distribution) from the downloads site.

3) Install a JCR instance to create a repository (Jackrabbit from Apache is the recommendation). To install Jackrabbit: download the jcr-instance-jar-with-dependencies.jar into an execution working directory (JCR_HOME); edit the jcr-instance.properties file and copy the file into the JCR_HOME.

4) Start the repository by executing the jar using "java – jar jcr-instance-jar-with-dependencies.jar jcr-instance.xml".

5) Copy the addama-rest.war file into your tomcat/webapps directory and then fill in the addama-rest.properties file and copy it to your tomcat/libs directory.

6) Copy the addama-html.war file into your tomcat/webapps directory, make sure the addama-rest war file started up properly first.

7) In your browser go to 

## Available tools

The following tools are available from the main download site.

• *REST/URI Extensions*. This is the main set of services that must be installed before any of the additional tools. The services allow for the retrieval of content using a URI.

• *JCR Browsing System*. A javascript/json/ajax based web application for browsing and editing the contents of a JCR instance. This is included as part of the main distribution.

• *Access Components*: Components are provided to support the development of applications that require JCR retrieving, searching and publishing functionality. This are provided as a separate download and code is provided for R, Matlab, Perl, Python, Java and Ruby.

• *Data Feeder*. A utility for loading a JCR from a sample tracking system. This utility demonstrates how annotations and data items can be mapped into a repository; and how simple monitoring can be used to mirror the contents of an existing sample tracking system in the JCR. This is built against the freely available SlimArray sample tracking software [12].

• *File Loader*. A utility for loading file data directly into a JCR instance using XPATH information to define the position. This runs as an executable Java jar.

## Authors' contributions

JB designed the system, managed the development team, and drafted the manuscript. HR worked on the design and implementation of the REST interfaces, the aggregation system and the portal. CC contributed to the manuscript, provided implementations for key services, and standardised the metadata for the services. DB principally worked on the analysis pipelines and associated services, and also contributed significantly towards the design. SK worked on the pipelines and web applications, and also project managed the team. IS instigated and guided the project. All authors read and approved the manuscript.
